# Infective Endocarditis Due to Q Fever From a Tick Bite: A Case Report

**DOI:** 10.7759/cureus.64075

**Published:** 2024-07-08

**Authors:** Fawaz Mohammed, Evan Gleaves, Patricia Tellez Watson, Heather Lusby, Jacqueline Dawson Dowe

**Affiliations:** 1 Internal Medicine, University of Kentucky College of Medicine, Bowling Green, USA; 2 Cardiology, University of Kentucky, Bowling Green, USA; 3 Infectious Diseases, The Medical Center at Bowling Green, Bowling Green, USA; 4 Cardiology, University of Kentucky College of Medicine, Bowling Green, USA

**Keywords:** culture-negative infective endocarditis, q fever, tick bite, mitral valve vegetation, coxiella burnetti infection

## Abstract

Infection from *Coxiella burnetti* causes Q fever that manifests with vague symptoms. We report a case of an individual admitted to the hospital with recurrent fevers with a history of multiple tick bites. Further workup revealed examination and laboratory findings consistent with Q fever endocarditis. Fevers resolved with doxycycline and hydroxychloroquine. Our case highlights that suspicion for Q fever should be maintained in patients presenting to the hospital with fevers of unknown origin for prompt recognition and appropriate treatment.

## Introduction

Q fever is a zoonotic disease caused by the intracellular gram-negative bacterium *Coxiella burnetii*. The occurrence of Q fever across the globe has been difficult to gauge as it is rarely reported. According to epidemiological data, it should be considered a notifiable disease in many countries [1]. Domesticated animals act as the human reservoir and infection is transmitted via inhalation of the bacterium from an infected animal. It is more prevalent among farmers and veterinary doctors and therefore regarded as an occupational disease [2]. Direct person-to-person transmission is very rarely seen [3]. Infection can be acute or chronic, with the former presenting with influenza-like symptoms [4]. The more serious form of the disease, chronic Q fever, can be seen in individuals with preexisting native valve disease which can manifest years after the initial infection [5]. Here, we report a case of *C. burnetii *endocarditis in an individual admitted to the hospital with fevers of unknown origin with no prior apparent exposures.

## Case presentation

A 66-year-old Albanian female with a past medical history of chronic kidney disease stage IIIa, prior pulmonary embolism, paroxysmal atrial fibrillation on apixaban 5 mg twice a day, hypothyroidism, essential hypertension, type 2 diabetes mellitus, frequent falls, and left knee replacement presented to the emergency department (ED) with shortness of breath, cough, and right greater than left leg pain of one-day duration.

**Figure 1:**
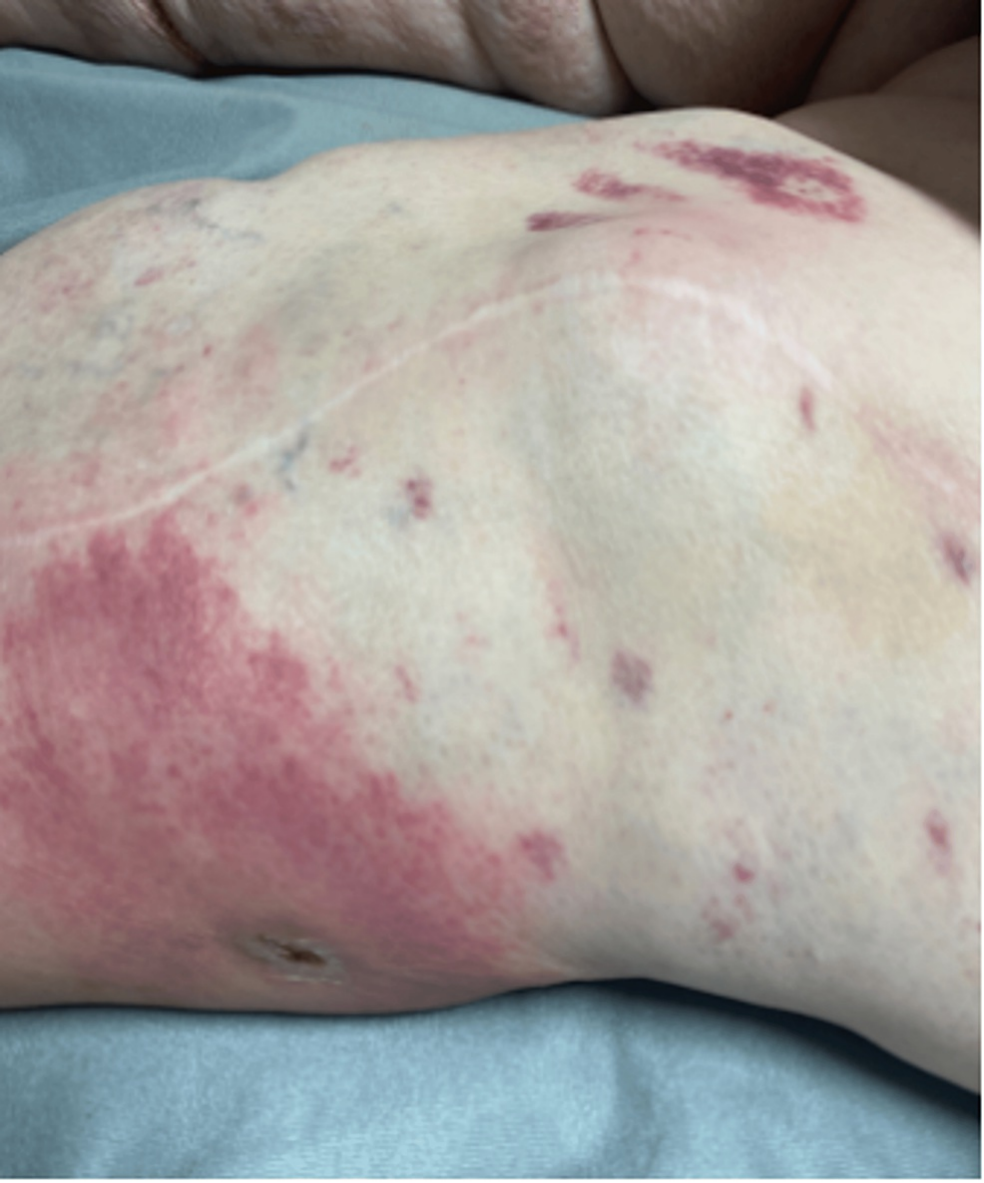
Left knee showing evidence of erythema.

**Figure 2:**
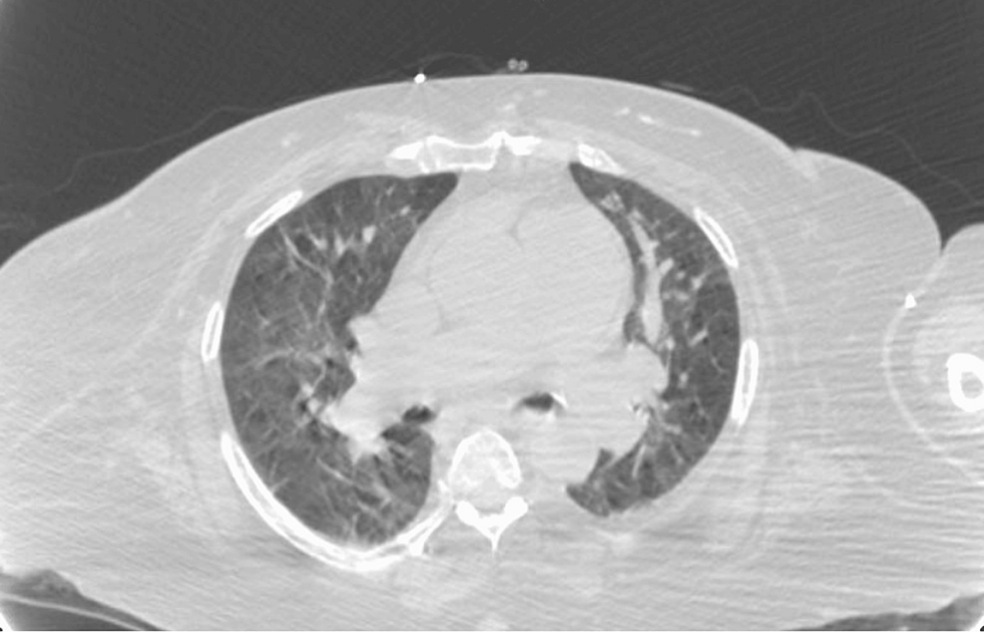
Computed tomography of the chest with evidence of bilateral opacities and pulmonary edema consistent with volume overload.

**Figure 3:**
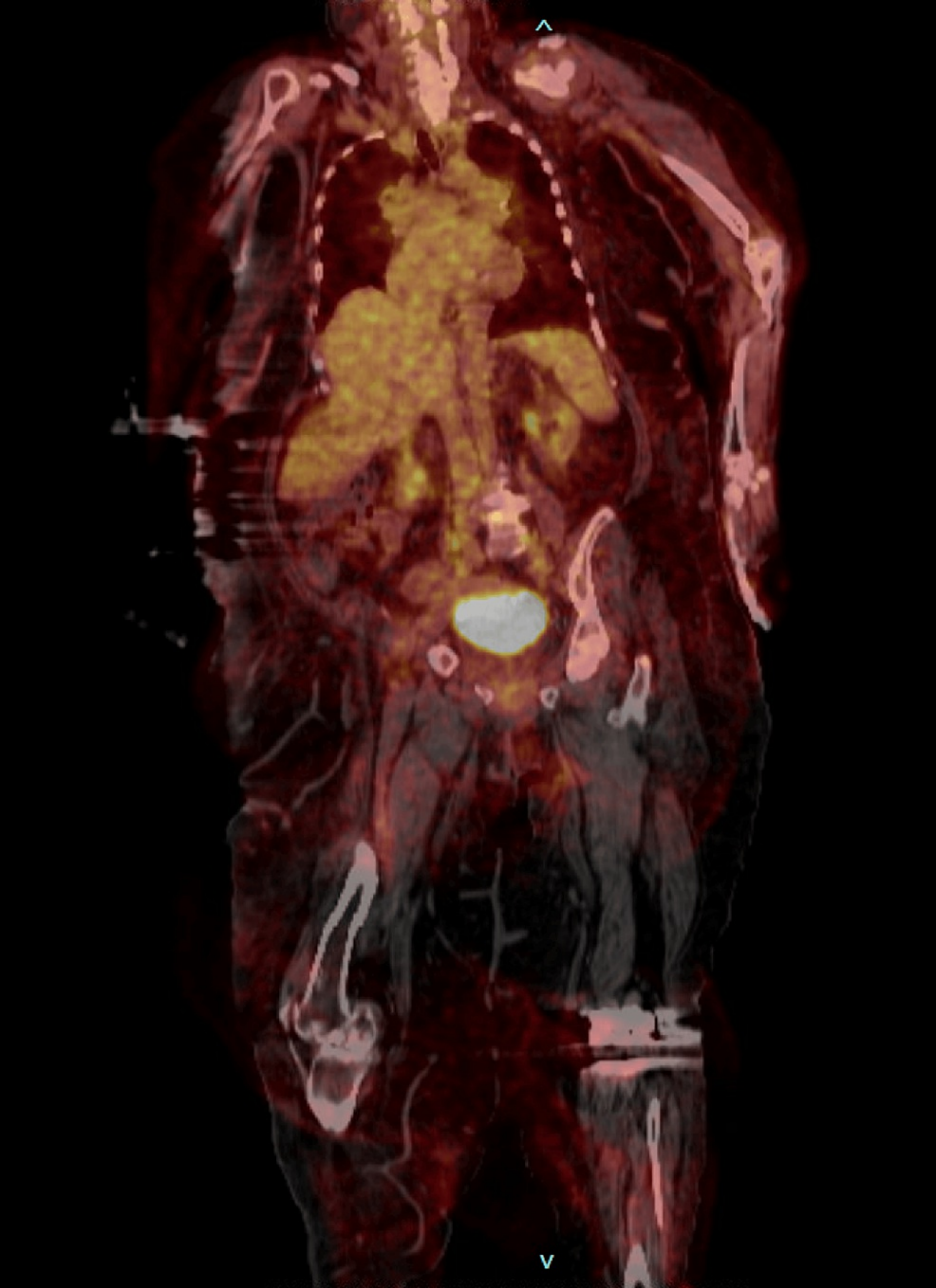
Positron emission tomography-computed tomography with no evidence of hypermetabolic activity.

In the ED, she was noted to have a fever of up to 104.3°F. Upon further questioning, the patient reported having fevers at home, particularly at night, for several weeks which she attributed to working hard around the house. A physical examination revealed the presence of multiple tick bites which the patient stated had occurred; however, she did not know the species or when exactly the bites occurred. Laboratory workup revealed leukocytosis with white blood cell count of 26.2 k/µL (4.8-10.8 k/µL), elevated erythrocyte sedimentation rate (ESR) of 57 mm/hour, C-reactive protein of 4.4 mg/dL (0-0.30 mg/dL), and lactic acid of 3.5 mmol/L (0.7-2 mmol/L). The left knee was erythematous, warm to the touch, and tender to palpation (Figure 1). Computed tomography (CT) of the chest obtained for shortness of breath revealed bilateral ground-glass opacities consistent with pulmonary edema (Figure 2). Volume overload improved with diuresis. She was empirically started on dalbavancin to cover for skin and soft tissue infection and possible septic joint. However, her fevers persisted and a positron emission tomography-computed tomography scan (PET-CT) performed to evaluate the source of the fevers showed no evidence of abnormal activity (Figure 3).

**Figure 4:**
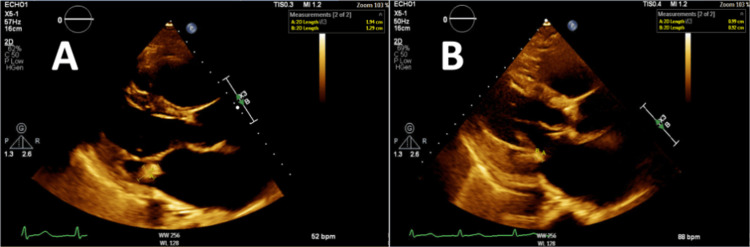
Transthoracic echocardiogram findings. (A) Parasternal long-axis view showing a 1.94 × 1.29 cm vegetation (arrows) on the mitral valve. (B) Parasternal long-axis view with the use of doxycycline and hydroxychloroquine demonstrating a decrease in the size of vegetation to 0.99 × 0.92 cm (arrows).

Joint infection was also ruled out given there was no hypermetabolic activity at the level of the left knee; therefore, joint aspiration was not pursued. Doxycycline was added with persistent fevers and suspicion for tick-borne infection with her history of multiple tick bites. On hospital day three, a tick-borne illness blood panel was sent including *Ehrlichia*, *Rickettsia*, *Lyme*, and *C. burnetii *polymerase chain reaction. Rapid plasma reagin and malaria smear were completed as well. Q fever immunoglobulin G (IgG) anti-phase II antibody titer was positive at >1:128 (1:16), suggestive of Q fever, with positive single convalescent serum sample in a patient who had been ill greater than one week in the absence of an acute sample. Blood cultures were negative. All other tick-borne illness testing was negative. A transthoracic echocardiogram (TTE) was performed to assess for infective endocarditis which showed a 1.94 × 1.29 cm vegetation on the mitral valve with moderate-to-severe mitral regurgitation, no evidence of pericardial effusion (Figure 4A). With continued doxycycline and hydroxychloroquine, the patient showed a resolution of fevers. With improvement in her heart failure symptoms following the initiation of medical therapy, surgery for infective endocarditis was not pursued. The patient was subsequently discharged. She was planned for a treatment course of 18 months with repeat *C. burnetii* serology every two months. She had a follow-up appointment after the initial two months of tolerating therapy and was found to have a decrease in* C. burnetii* phase II IgG antibody at 1:32, and her phase I IgG antibody was noted to be negative. Due to multiple recent hospitalizations, she has not been able to follow up as an outpatient with infectious disease for repeat titers but is still on doxycycline and hydroxychloroquine. A follow-up TTE performed five months following treatment initiation with doxycycline and hydroxychloroquine showed a decrease in the size of vegetation to 0.99 × 0.92 cm (Figure 4B). The patient has been on doxycycline and hydroxychloroquine for 10 months with a tentative end date of therapy in February 2025 depending upon the clinical course and repeat titers.

## Discussion

Epidemiological data have shown that Q fever infection has been reported worldwide given the ubiquity of the causative organism. A review of the literature has suggested that *C. burnetii* infections are typically seen as standalone cases or a group of cases occurring as a part of an outbreak [6]. Domesticated animals are often the reservoir of infection with recent investigations reporting an increasing number of cases with dogs and cats acting as the reservoir of the infection [7].

Infection in humans is acquired from inhalation of the organism when it is dissipated in air from infected products (fomites, products of conception). In the acute form of the disease, more than 50% of the cases are asymptomatic, with symptomatic individuals presenting with vague non-specific symptoms (fever, cough, myalgia) [8]. A small percentage of individuals go on to develop chronic infection seen classically in patients with preexisting valvular abnormalities or immunocompromised states, with one study suggesting that approximately 39% of individuals who develop acute infection and have a preexisting valvular disorder tend to develop endocarditis [9-11]. More than 70% of these cases are seen in men, with active cancer being the most common risk factor for developing endocarditis [12,13]. Numerous cases have also been described in individuals with acquired immunodeficiency syndrome [14]. Our patient was a female with no history of autoimmune disease or known malignancy, which is typically seen in the disease. Our patient immigrated from Albania where the prevalence of Q fever is known to be higher [15]. On presentation, Q fever endocarditis usually lacks features of bacterial endocarditis which often delays the diagnosis. Houpikian et al. reported an average delay of about seven months from the onset of symptoms [12]. Endocarditis complicated by heart failure is a common presenting feature and fever is often not present in many cases [11]. One-third of cases have been shown to have embolic phenomena, although this is seen in the advanced form of the disease [16]. Microscopic hematuria secondary to immune complex deposits have also been reported [17]. About half of the cases can have hepatosplenomegaly with elevations in aspartate aminotransferase and alanine transaminase on blood chemistries [1]. Abnormalities of blood profile range from elevated ESR, anemia (seen in half of the individuals), and leukocytosis or leukopenia and thrombocytopenia seen in a few cases [9,12].

The initial diagnostic imaging modality for endocarditis, TTE, has limited utility in diagnosing chronic Q fever endocarditis largely due to the small size of vegetation in the cases [1,18]. Although transesophageal echocardiography is known to be superior to TTE, evidence has shown that it is of limited value [12].

Being an obligate intracellular organism, diagnosis is further hindered as it only grows on living cell lines and not routine culture media [19]. Diagnosis of chronic Q fever is through serological testing which characteristically shows high titers against phase I antigen, with an IgG anti-phase I antibody titers equal to 1:800 or greater yielding a specificity of 99.6% in diagnosing chronic Q fever [20-22]. Evidence of infective endocarditis on histopathology is the gold standard for diagnosing infective endocarditis where diagnosis can be challenging, with a study showing about 18% of cases only have typical features on histopathology [23]. Prognosis is generally poor thought to be due to delayed diagnosis which delays appropriate treatment. If caught early, the mortality rates may be as low as 10% [12].

The first choice of treatment for acute disease is doxycycline. Combination treatment with hydroxychloroquine and doxycycline is recommended for chronic Q fever. Patients require treatment for up to 18 months making them susceptible to side effects secondary to these medications.

To ensure the efficacy of the selected treatment regimen, phase I serologies should be obtained regularly. Phase I IgG titers less than 1:200 are the criteria for disease clearance [14]. Q fever also has high rates of relapse and patients should be closely monitored at regular intervals [1].

## Conclusions

Diagnosing Q fever is often challenging given the insidious nature of the disease with non-specific findings on presentation lacking typical findings of infective endocarditis. A high index of suspicion should be maintained in patients with previous valvular pathology and other risk factors presenting with fevers of unknown origin. Early recognition is warranted given the poor prognosis with delayed diagnosis and appropriate treatment.
